# Pseudoplasma of the Mouth

**Published:** 1858-07

**Authors:** 


*Pseudoplasma of the Mouth.*—We publish in the present number of the Journal, the first of a series of papers on the above named affection, from Dr. Cartier, a recent graduate of the Baltimore Dental College. That he has studied the etiology and pathology of the morbid productions of the buccal cavity with much care and attention, is evident from the manner in which he treats the subject. The hospital advantages which he enjoyed previously to coming to this country, afforded him abundant opportunities of studying diseases of this kind. We have no doubt, therefore, that the papers of Dr. Cartier will be read with pleasure and profit. The style in which they are written is a little stiff, and he may not always express himself as clearly as could be wished, but it must be recollected that he writes in a language which he has but recently learned, and we believe few foreigners have been able to acquire as correct a knowledge of the English language in one year, as he has done.

He sails on the 3d of the present month for France, where we believe he proposes to enter upon the practical duties of his profession. We wish him great success—that he is deserving of it, we are well assured.

